# Three new species of *Aleurodiscus* s.l. (Russulales, Basidiomycota) from southern China

**DOI:** 10.3897/mycokeys.37.25901

**Published:** 2018-08-03

**Authors:** Yan Tian, Masoomeh Ghobad-Nejhad, Shuang-Hui He, Yu-Cheng Dai

**Affiliations:** 1 Institute of Microbiology, Beijing Forestry University, Beijing 100083, China Beijing Forestry University Beijing China; 2 Department of Biotechnology, Iranian Research Organization for Science and Technology (IROST), Tehran 15819, Iran Iranian Research Organization for Science and Technology Tehran Iran; 3 Beijing Advanced Innovation Centre for Tree Breeding by Molecular Design, Beijing Forestry University, Beijing 100083, China Beijing Forestry University Beijing China

**Keywords:** acanthophyses, corticioid fungi, Stereaceae, taxonomy, wood-inhabiting fungi

## Abstract

Three new species of *Aleurodiscus* s.l. with corticioid basidiomata are described and illustrated from southern China based on morphological evidence and phylogenetic analyses of ITS and nrLSU sequence data. *Aleurodiscusbambusinus* was collected from Jiangxi Province on bamboo and is distinct by having a compact texture, simple-septate generative hyphae, abundant acanthophyses, basidia with acanthophysoid appendages and smooth basidiospores. *Aleurodiscusisabellinus* was collected from Yunnan Province on both angiosperm wood and bamboo and is distinct by having soft basidiomata with yellow to yellowish-brown hymenophore, yellow acanthophyses, simple-septate generative hyphae and smooth basidiospores. *Aleurodiscussubroseus* was collected from Guangxi Autonomous Region and Guizhou Province on angiosperm wood and is distinct by having pinkish basidiomata when fresh, clamped generative hyphae, clavate acanthophyses and echinulate basidiospores. In the phylogenetic tree, *A.bambusinus* and *A.isabellinus* were nested within the *A.cerussatus* group, whilst *A.subroseus* was clustered with *A.wakefieldiae*. An identification key to 26 species of *Aleurodiscus* s.l. in China is provided.

## Introduction

*Aleurodiscus* s.l. is a large group of wood-inhabiting fungi with a broad morphological circumscription. It is characterised by having cupulate, effused or effused-reflexed basidiomata, a monomitic or dimitic hyphal system with simple-septate or clamped generative hyphae, smooth or ornamented, amyloid basidiospores and sterile organs such as acanthophyses, gloeocystidia and dendrohyphidia (Núñez and Ryvarden 1997). Although *Aleurodiscus* s.l. had been divided into several small genera based on different combinations of morphological characters, phylogenetic analyses did not fully support these separations (Wu et al. 2001; Dai and He 2016). Accordingly, the inter- and intra-generic phylogeny of *Aleurodiscus* s.l. in Stereaceae is still unclear and no reliable morphological characters can be used to recognise the small segregated genera. Thus, the broad sense concept of the genus has often been adopted by mycologists when describing new species (Núñez and Ryvarden 1997; Gorjón et al. 2013; Dai et al. 2017a, b).

A recent survey on *Aleurodiscus* s.l. from China (Dai and He 2016, 2017, Dai et al. 2017a, b) revealed that its species diversity is high and many species, especially those with corticioid basidiomata on both herbaceous and ligneous plants, are still undescribed. In the present study, three new species are described and illustrated from southern China, amongst which two species have abundant acanthophyses and smooth basidiospores and one species bears echinulate basidiospores. Morphological differences between new species and their relatives are discussed. Their phylogenetic positions were inferred from a combined dataset of ITS and nrLSU sequence data.

## Materials and methods

### Morphological studies

Voucher specimens are deposited in the herbaria of Beijing Forestry University, Beijing, China (BJFC), Centre for Forest Mycology Research, U.S. Forest Service, Madison, USA (CFMR) and Southwest Forestry University, Kunming, China (SWFC). Freehand sections were made from basidiomata and mounted in 2% (w/v) potassium hydroxide (KOH), 1% phloxine (w/v) or Melzer’s reagent. Microscopic examinations were carried out with a Nikon Eclipse 80i microscope at magnifications up to 1000×. Drawings were made with the aid of a drawing tube. The following abbreviations are used: L = mean spore length, W = mean spore width, Q = L/W ratio, n (a/b) = number of spores (a) measured from number of specimens (b). Colour names and codes follow Kornerup and Wanscher (1978).

### DNA extraction and sequencing

A CTAB plant genome rapid extraction kit-DN14 (Aidlab Biotechnologies Co. Ltd, Beijing) was employed for DNA extraction and PCR amplification from dried specimens. The ITS and nrLSU gene regions were amplified with primer pairs ITS5/ITS4 (White et al. 1990) and LR0R/LR7 (http://www.biology.duke.edu/fungi/mycolab/primers.htm), respectively. The PCR procedures followed Dai and He (2016). DNA sequencing was performed at Beijing Genomics Institute and the sequences were deposited in GenBank.

### Phylogenetic analyses

The molecular phylogeny was inferred from a combined dataset of ITS and nrLSU sequences of representative members of Stereaceae sensu Larsson (2007) (Table 1). The ingroup taxa sampling and outgroup selection followed Dai et al. (2017b). The sequences were aligned using MAFFT v.6 (Katoh and Toh 2008, http://mafft.cbrc.jp/alignment/server/). Alignments were optimised manually in BioEdit 7.0.5.3 (Hall 1999) and deposited at TreeBase (http://treebase.org/treebase-web/home.html, submission ID: 22474). Maximum Parsimony (MP), Bayesian Inference (BI) and Maximum Likelihood (ML) analyses were performed by using PAUP* 4.0b10 (Swofford 2002), MrBayes 3.1.2 (Ronquist and Huelsenbeck 2003) and RAxML 7.2.6 (Stamatakis 2006), respectively. The best models of evolution for BI were estimated by using MrModeltest 2.2 (Nylander 2004). The methods and parameter settings for the three kinds of phylogenetic analyses followed Liu et al. (2018).

**Table 1.** Species and sequences used in the phylogenetic analyses. Newly generated sequences are set in bold.

Taxa	Voucher	Locality	ITS	nrLSU
* Acanthobasidium bambusicola *	He 2357	China	KU559343	KU574833
* A. norvegicum *	T 623	France	–	AY039328
* A. phragmitis *	CBS 233.86	France	–	AY039305
* A. weirii *	HHB 12678	USA	–	AY039322
* Acanthofungus rimosus *	Wu 9601-1	Taiwan	–	AY039333
* Aleurodiscus abietis *	T 330	Canada	–	AY039324
* A. amorphus *	Ghobad-Nejhad 2464	China	KU559342	KU574832
* A. aurantius *	T 621	France	–	AY039317
*** A. bambusinus ***	**He 4261**	**China**	**KY706207**	**KY706219**
*** A. bambusinus ***	**He 4263**	**China**	**KY706208**	**KY706218**
* A. bisporus *	T 627	Guadeloupe	–	AY039318
* A. botryosus *	He 2712	China	KX306877	KY450788
* A. canadensis *	Wu 1207-90	China	KY706203	KY706225
* A. cerussatus *	He 2208	China	KX306874	KY450785
* A. dextrinoideocerussatus *	He 2820	China	KY706206	MH109044
* A. dextrinoideophyses *	He 4105	China	MH109050	KY450784
* A. effusus *	He 2261	China	KU559344	KU574834
* A. gigasporus *	Wu 0108-15	China	KY706205	KY706213
* A. grantii *	He 2895	China	KU559347	KU574837
*** A. isabellinus ***	**He 5283**	**China**	**MH109052**	**MH109046**
*** A. isabellinus ***	**He 5294**	**China**	**MH109053**	**MH109047**
* A. lapponicus *	FP 100753	USA	–	AY039320
* A. lividocoeruleus *	MB 1825	USA	–	AY039314
* A. mesaverdensis *	FP 120155	USA	KU559359	KU574817
* A. mirabilis *	Dai 13281	China	KU559350	KU574839
* A. oakesii *	He 2243	USA	KU559352	KU574840
* A. penicillatus *	HHB 13223	USA	–	KU574816
***A.* sp.**	**Ghobad-Nejhad 2360**	**China**	**MH109051**	**MH109045**
*** A. subroseus ***	**He 4807**	**China**	**MH109054**	**MH109048**
*** A. subroseus ***	**He 4814**	**China**	**MH109055**	**MH109049**
* A. tenuissimus *	He 3575	China	KX306880	KX842529
* A. thailandicus *	He 4099	Thailand	KY450781	KY450782
* A. tropicus *	He 3830	China	KX553875	KX578720
* A. tropicus *	He 3834	China	KX553876	KY706221
* A. verrucosporus *	He 4491	China	KY450786	KY450790
* A. wakefieldiae *	He 2580	China	KU559353	KU874841
* Boidinia macrospora *	Wu 9202-2	China: Taiwan	AF506377	AF506377
* Conferticium heimii *	CBS 321.66	Central African Republic	AF506381	AF506381
* C. ravum *	NH 13291	Estonia	AF506382	AF506382
* Gloeocystidiellum aspellum *	LIN 625	China: Taiwan	AF506432	AF506432
* Gloeocystidiopsis cryptacanthus *	KHL 10334	Puerto Rico	AF506442	AF506442
* G. flammea *	AH 000219	La Réunion	AF506438	AF506438
* Gloeodontia discolor *	KHL 10099	Puerto Rico	AF506445	AF506445
* G. pyramidata *	LR 15502	Columbia	AF506446	AF506446
* Megalocystidium chelidonium *	LodgeSJ 110.1	USA	AF506441	AF506441
*M.leucoxanthu*m	HK 82	Denmark	AF506420	AF506420
* M. wakullum *	Oslo 930107	Tanzania	AF506443	AF506443
* Neoaleurodiscus fujii *	He 2921	China	KU559357	KU574845
* Stereum complicatum *	He 2234	USA	KU559368	KU574828
* S. ostrea *	He 2067	USA	KU559366	KU574826
* S. sanguinolentum *	He 2111	USA	KU559367	KU574827
* Xylobous frustulatus *	He 2231	USA	KU881905	KU574825
* X. subpileatus *	FP 106735	USA	–	AY039309

### Phylogeny results

**Figure 1.**
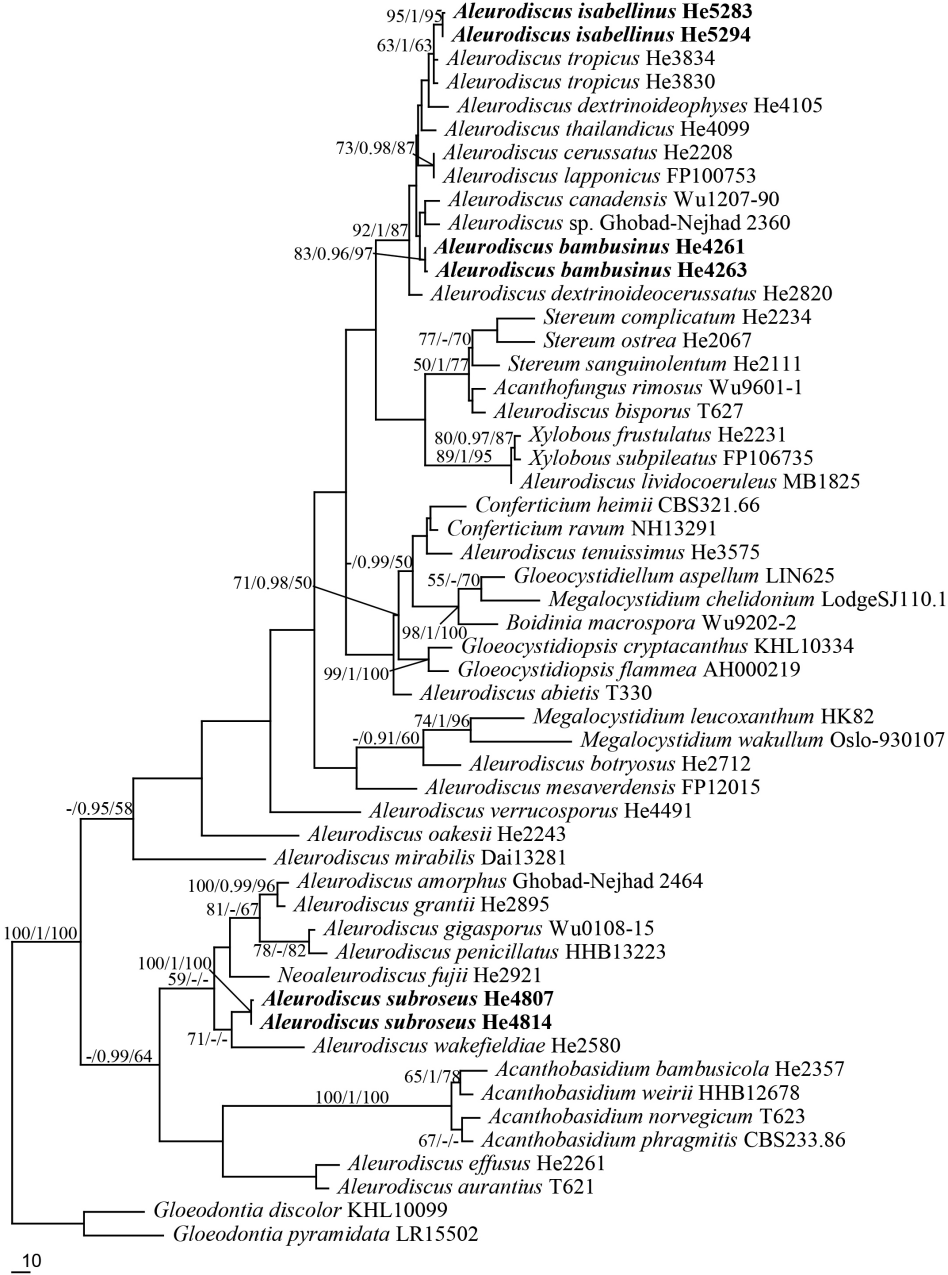
Maximum parsimony phylogeny of the combined ITS and nrLSU sequences data of Stereaceae. Branches are labelled with maximum parsimony and maximum likelihood bootstrap values ≥50% and Bayesian posterior probabilities ≥0.95 (MP/BI/ML).

The ITS-nrLSU sequences dataset contained 42 ITS and 53 nrLSU sequences from 53 samples representing 47 ingroup taxa and the outgroup (Table 1). Seven ITS and seven nrLSU sequences were generated for this study. The dataset had an aligned length of 2045 characters, of which 384 were parsimony informative. Maximum Parsimony (MP) analysis yielded 85 equally parsimonious trees. The best model estimated and applied in the Bayesian analysis was GTR+I+G. The average standard deviation of split frequencies of BI was 0.007863. ML and BI analyses resulted in almost the same tree topologies as that of MP analysis. Only the MP tree is shown in Fig. 1 with maximum likelihood and maximum parsimony bootstrap values ≥50% and BPP ≥0.95 labelled along the branches. In the tree, *A.bambusinus* and *A.isabellinus* were nested within the *A.cerussatus* (Bres.) Höhn. & Litsch. group (MP = 92%, BI = 1.00, ML = 87%). *Aleurodiscussubroseus* was clustered with *A.wakefieldiae*, but their relationship has no support in BI and ML analyses.

## Taxonomy

### Aleurodiscus bambusinus

Taxon classificationFungiRussulalesStereaceae

S.H. He & Y.C. Dai sp. nov.

824755

Figs 2a–b , 3

#### Diagnosis.

The species is distinct by having corticioid basidiomata, a compact texture, simple-septate generative hyphae, abundant acanthophyses, basidia with an acanthophysoid appendage and smooth basidiospores 7–10 × 4–6 μm and growing on bamboo.

#### Holotype.

CHINA. Jiangxi Province, Yifeng County, Guanshan Nature Reserve, alt. ca. 800 m, on fallen culms and branches of bamboo, 10 Aug 2016, He 4261 (holotype, BJFC 023703).

#### Etymology.

“*Bambusinus*” refers to the substrate of bamboo.

#### Basidiomata.

Annual, resupinate, effused, closely adnate, inseparable from substrate, coriaceous, at first as small patches, later confluent up to 30 cm long and 2.5 cm wide, 180–300 μm thick. Hymenophore smooth, white (4A1) to yellowish-white (4A2) when young, becoming greyish-yellow [4B (3–4)] to brownish-orange [6C (5–8)] with age, uncracked or cracked with age; margin abrupt, indistinct, concolorous with hymenophore.

#### Microscopic structures.

Hyphal system monomitic; generative hyphae simple-septate, colourless, thin- to thick-walled, scattered near the substrate, 2–4 μm in diam. Subiculum thin to indistinct. Subhymenium thick, with compact texture, composed of acanthophyses and gloeocystidia. Acanthophyses abundant, hyphoid or distinctly swollen in the middle part, colourless, thin-walled, with abundant spines in apex, 30–40 × 3–12 μm. Gloeocystidia abundant, flexuous or slightly moniliform with one to several constrictions, slightly thick-walled, negative in sulphobenzaldehyde, 30–55 × 8–13 μm. Basidia subclavate to subcylindrical, colourless, slightly thick-walled, usually with a lateral acanthophysoid appendage, with four sterigmata and a basal simple septum, 25–35 × 7–9 μm. Basidiospores ellipsoid to broadly ellipsoid, bearing a distinct apiculus, colourless, thin-walled, smooth, amyloid, 7–10 × 4–6 μm, L = 8.7 μm, W = 4.9 μm, Q = 1.6–1.9 (n = 90/3).

#### Additional specimens examined.

CHINA. Jiangxi Province, Yifeng County, Guanshan Nature Reserve, alt. ca. 800 m, on fallen culms and branches of bamboo, 10 Aug 2016, He 4250 (BJFC 023692) and He 4263 (BJFC 023705).

#### Remarks.

*Aleurodiscusbambusinus* is morphologically similar and phylogenetically close to *A.dextrinoideophyses* S.H. He and *A.tropicus* L.D. Dai & S.H. He that also grow on bamboo in East Asia (Dai et al. 2017a, b). *Aleurodiscusdextrinoideophyses* differs from *A.bambusinus* by having apparently dextrinoid acanthophyses and smaller basidiospores (5–7 × 3–4 μm, Dai et al. 2017b). *Aleurodiscustropicus* differs from *A.bambusinus* by having a looser texture and slightly larger basidiospores (9–12 × 5–7.5 μm, Dai et al. 2017a). The ITS similarity between *A.bambusinus* (He 4261) and *A.dextrinoideophyses* (He 4105) is 95.6% of 434 base pairs and, between *A.bambusinus* (He 4261) and *A.tropicus* (He 3830), is 97.3% of 582 base pairs. *Aleurodiscusaberrans* G. Cunn. and *A.rimulosus* Núñez & Ryvarden are also similar to *A.bambusinus*, but they differ from this new species by having smooth basidia and growing on angiosperm wood outside of Asia (Núñez and Ryvarden 1997).

**Figure 2.**
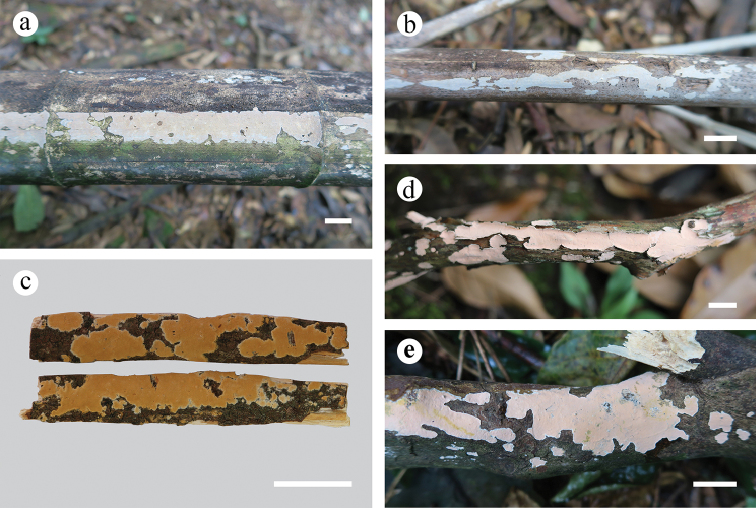
Basidiomata. **a–b***Aleurodiscusbambusinus* (**a** He 4250 **b** holotype, He 4261) **c***A.isabellinus* (holotype, KKN-2017-19) **d–e***A.subroseus* (**d** He 5571 **e** He 4895). Scale bars: 1 cm.

**Figure 3.**
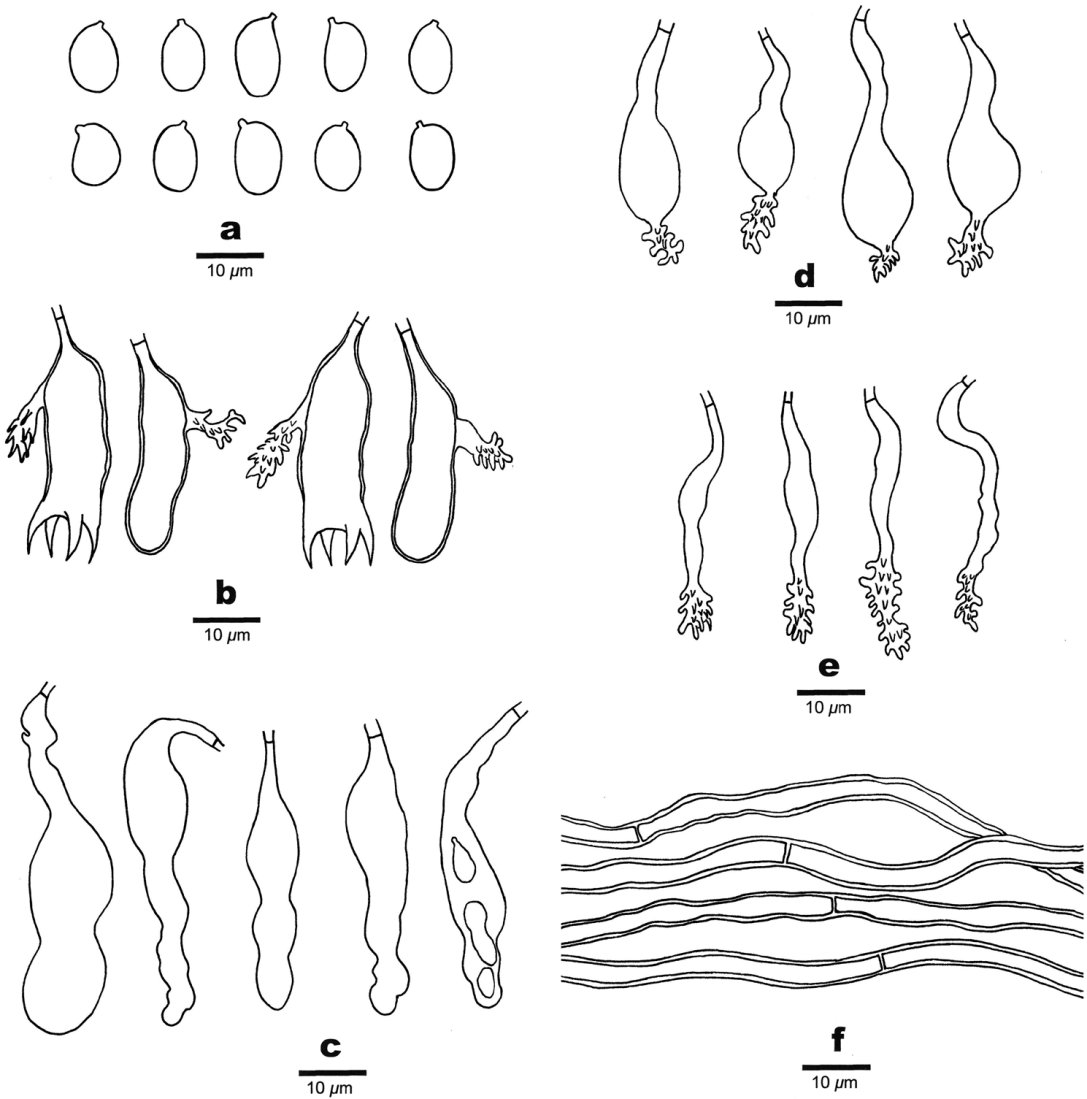
Microscopic structures of *Aleurodiscusbambusinus* (drawn from the holotype). **a** Basidiospores; **b** Basidia **c** Gloeocystidia **d–e** Acanthophyses **f** Generative hyphae.

### Aleurodiscus isabellinus

Taxon classificationFungiRussulalesStereaceae

S.H. He & Y.C. Dai sp. nov.

824758

Figs 2c , 4

#### Diagnosis.

The species is distinct by having soft, yellow to yellowish-brown and corticioid basidiomata, a loose texture, abundant yellow acanthophyses, simple-septate generative hyphae and smooth basidiospores 6–8.5 × 3–4 μm.

#### Holotype.

CHINA. Yunnan Province, Dali County, Cangshan Nature Reserve, alt. ca. 2600 m, on fallen decorticated angiosperm branches, 27 Oct 2017, KKN-2017-19 (holotype in CFMR, isotype in BJFC).

#### Etymology.

“*Isabellinus*” refers to the yellowish-brown basidiomata.

#### Basidiomata.

Annual, resupinate, effused, adnate, inseparable from substrate, soft, membranaceous to coriaceous, at first as small patches, later confluent up to 15 cm long and 1 cm wide, 150–300 μm thick. Hymenophore smooth, light orange [5A(4–5)] , greyish-orange[5B(5–6)], orange [5B(7–8)] to brownish-yellow [5C(7–8)], uncracked or cracked with age; margin thinning out, fimbriate, white (5A1) when juvenile, becoming abrupt, indistinct, concolorous with hymenophore when mature.

#### Microscopic structures.

Hyphal system monomitic, generative hyphae simple-septate, colourless, thin- to slightly thick-walled, straight, loosely interwoven, frequently branched and septate, 2–4 μm in diam. Acanthophyses abundant, colourless to yellow, thick-walled, hyphoid or arising laterally or apically from a clavate or cylindrical base 30–50 × 5–7 μm, with abundant spines in upper part, some hyphoid ones near substrate with long spines (branches) resembling binding hyphae. Gloeocystidia abundant, embedded, colourless, slightly thick-walled, subcylindrical or slightly moniliform, negative in sulphobenzaldehyde, 35–110 × 5–8 μm. Basidia clavate, colourless, thin-walled, with four sterigmata and a basal simple septum, 40–55 × 6–7 μm. Basidiospores ellipsoid to oblong ellipsoid, bearing a distinct apiculus, colourless, thin-walled, smooth, amyloid, (5.5–) 6–8.5 × (2.8–) 3–4 μm, L = 7 μm, W =3.7 μm, Q = 1.9 (n = 24/1).

#### Additional specimens examined.

CHINA. Yunnan Province, Dali County, Cangshan Nature Reserve, alt. ca. 2600 m, on small dead bamboo, 27 Oct 2017, He 5283 (BJFC 024801) and He 5287 (BJFC 024805); on fallen angiosperm branch, 27 Oct 2017, He 5294 (BJFC 024812); Jingdong County, Ailaoshan Nature Reserve, alt. 2450 m, on fallen angiosperm branch, 4 Oct 2017, C.L. Zhao 3843 (SWFC).

#### Remarks.

All the studied specimens of *A.isabellinus* lack a true hymenium and only the holotype has a few basidia and basidiospores. *Aleurodiscusisabellinus* was nested within the *A.cerussatus* group (Fig. 1). In this group, *Aleurodiscusthailandicus* S.H. He is similar to *A.isabellinus* by sharing the yellow basidiomata and acanthophyses, but differs by having two types of gloeocystida and acanthophyses without a clavate or cylindrical base (Dai et al. 2017a). The ITS similarity between *A.isabellinus* (He 5283) and *A.thailandicus* (He 4099) is 93.6% of 578 base pairs. *Aleurodiscusthailandicus* was described from Thailand based on a fertile specimen on bamboo, but later several sterile specimens on bamboo from south-western China were identified as this species according to the sequence data. Morphologically, the soft and yellow to yellowish-brown basidiomata of *A.isabellinus* resemble the genus *Vararia* P. Karst. which belongs to Peniophoraceae according to phylogenetic analyses.

**Figure 4.**
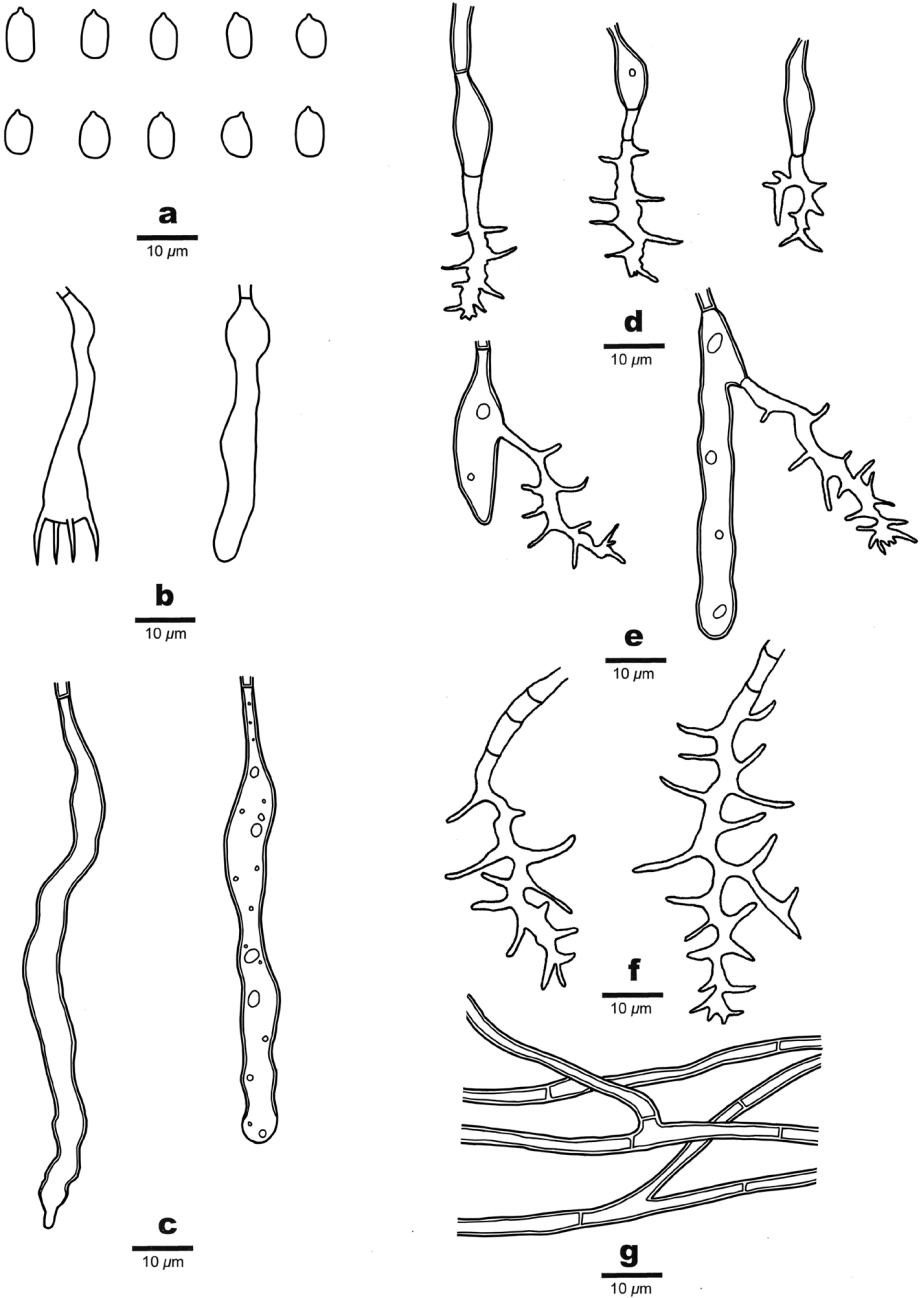
Microscopic structures of *Aleurodiscusisabellinus* (drawn from the isotype). **a** Basidiospores **b** A basidium and a basidiole **c** Gloeocystidia **d–f** Acanthophyses **g** Generative hyphae.

### Aleurodiscus subroseus

Taxon classificationFungiRussulalesStereaceae

S.H. He & Y.C. Dai sp. nov.

824757

Figs 2d–e , 5

#### Diagnosis.

The species is distinct by having pinkish and corticioid basidiomata when fresh, clamped generative hyphae, moniliform gloeocystidia, presence of acanthophyses (acanthocystidia) and echinulate basidiospores 16–20 × 11–14 μm.

#### Holotype.

CHINA. Guangxi Autonomous Region, Xing’an County, Mao’ershan Nature Reserve, alt. ca. 1600 m, on dead but still attached branch of living angiosperm tree, 13 Jul 2017, He 4807 (holotype, BJFC 024326).

#### Etymology.

“*Subroseus*” (Lat.) refers to the pinkish basidiomata when fresh.

#### Basidiomata.

Annual, resupinate, effused, closely adnate, inseparable from substrate, coriaceous, at first as small irregular patches, later confluent up to 35 cm long and 3 cm wide, up to 300 μm thick. Hymenophore smooth, pinkish-white (12A2), pink (12A3), pale orange (6A3) to light orange (6A4) when fresh, becoming pale orange (6A3), light orange [6A(4–5)], greyish-orange [6B(3–6)] to brownish-orange [6C(5–6)] when dry, uncracked; margin abrupt, white and distinct when fresh, becoming concolorous or darker than hymenophore and indistinct when dry, slightly elevated when mature.

#### Microscopic structures.

Hyphal system monomitic, generative hyphae with clamp connections. Subiculum thin to indistinct. Subhymenium thickening with age, with embedded gloeocystidia, acanthophyses and crystals. Hyphae in this layer colourless, thin-walled, frequently branched and septate, agglutinated, 2–4 μm in diam. Gloeocystidia abundant, moniliform, with one to several constrictions, smooth, slightly thick-walled, negative in sulphobenzaldehyde, 45–70 × 6–12 μm. Acanthophyses (acanthocystidia) abundant, variable in shape and size, subclavate to subcylindrical, with few to many spines at apex, colourless, slightly thick-walled, 30–60 × 6–20 μm. Hyphidia scattered, thin-walled, colourless, rarely branched. Basidia clavate, slightly sinuous, colourless, thin-walled, smooth, with four sterigmata and a basal clamp connection, 52–80 × 13–17 μm. Basidiospores ellipsoid to broadly ellipsoid, bearing a distinct apiculus, colourless, slightly thick-walled, echinulate, strongly amyloid, 16–20 × 11–14 μm, L = 18.4 μm, W = 12.6 μm, Q = 1.5 (n = 90/3) (spines excluded).

#### Additional specimens examined.

CHINA. Guangxi Autonomous Region, Xing’an County, Mao’ershan Nature Reserve, alt. ca. 1600 m, on dead but still attached branch of living angiosperm tree, 13 Jul 2017, He 4814 (BJFC 024333); Jinxiu County, Dayaoshan Nature Reserve, Yinshan Forest Park, alt. ca. 1500 m, on fallen angiosperm branch, 16 Jul 2017, He 4895 (BJFC 024414). Guizhou Province, Jiangkou County, Fanjingshan Nature Reserve, alt. 1500–2000 m, on dead but still attached branch of living angiosperm tree, 11 Jul 2018, He 5558 (BJFC); 12 Jul 2018, He 5571, He 5577, He 5581, He 5585, He 5589 and He 5593 (BJFC).

#### Remarks.

*Aleurodiscussubroseus* is morphologically similar and phylogenetically close to *A.wakefieldiae* Boidin & Beller (Fig. 1), but the latter differs by having longer basidia (80–180 μm) and larger basidiospores (20–28 × 14–20 μm, Núñez and Ryvarden 1997). *Aleurodiscuspenicillatus* Burt is similar to *A.subroseus*, but differs by growing on gymnosperm wood and having wider basidiospores (13–17 μm, Núñez and Ryvarden 1997). *Aleurodiscusmirabilis* (Berk. & M.A. Curtis) Höhn. also has pinkish fresh basidiomata and is widely distributed in southern China. However, it can be easily distinguished from *A.subroseus* by having basally warted basidia and larger basidiospores (24–28 × 14–17 μm, Núñez and Ryvarden 1997). In the phylogenetic tree (Fig. 1), *A.penicillatus* and *A.mirabilis* are distantly related to *A.subroseus*. *Aleurodiscuscorticola* Gorjón et al. from Argentina on bark of living *Nothofagusdombeyi* also has moniliform gloeocystidia and similar basidiospores with *A.subroseus*, but differs by having pulvinate and tuberculate basidiomata and absence of acanthophyses (Gorjón et al. 2013).

**Figure 5.**
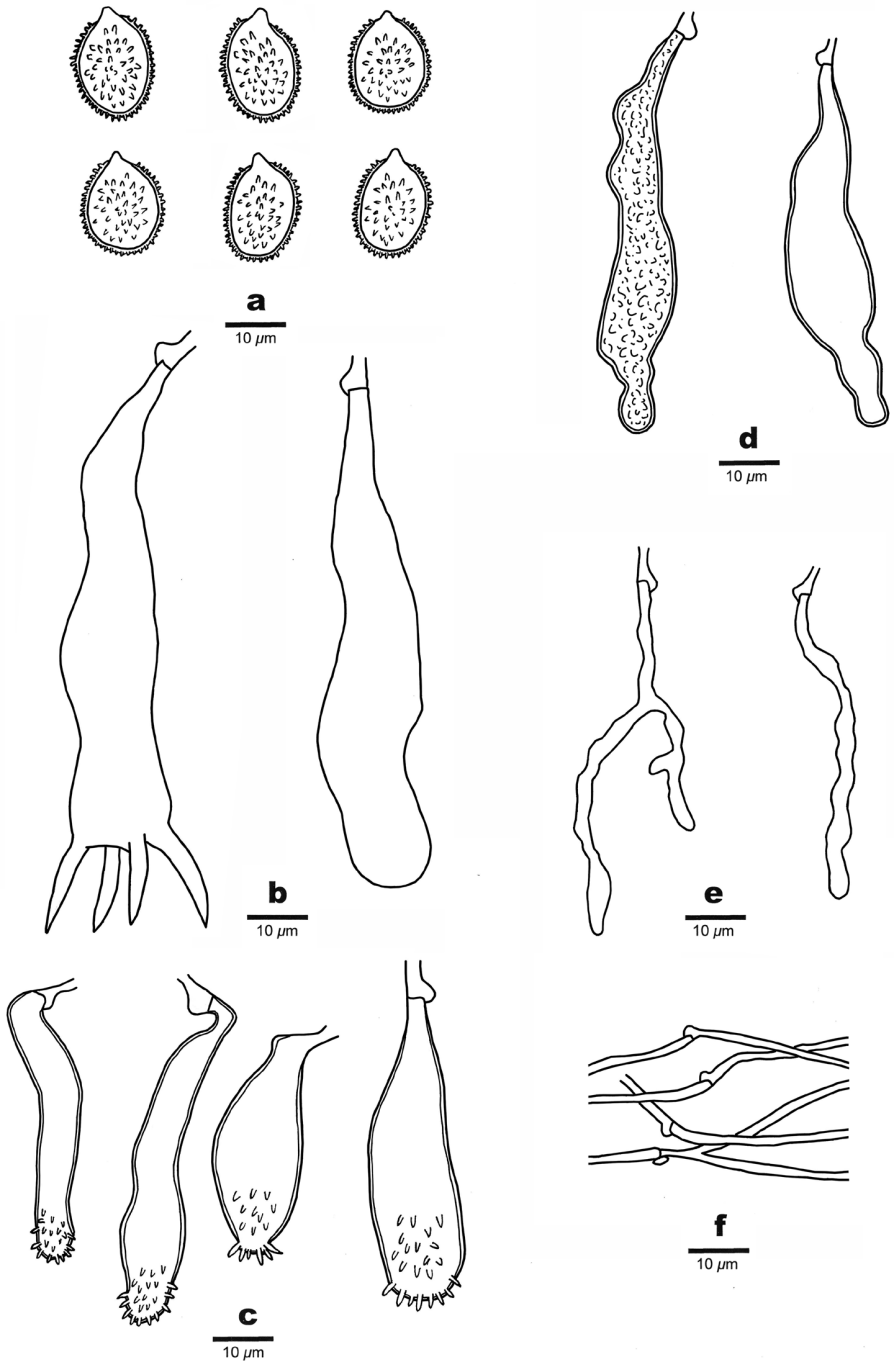
Microscopic structures of *Aleurodiscussubroseus* (drawn from the holotype). **a** Basidiospores; **b** A basidium and a basidiole **c** Acanthophyses **d** Gloeocystidia **e** Hyphidia **f** Generative hyphae.

### Key to 26 species of *Aleurodiscus* s.l. in China

*Acanthobasidium* Oberw., *Aleurocystidiellum* P.A. Lemke and *Neoaleurodiscus* Sheng H. Wu are used for some species. Basidiospores data are from Núñez & Ryvarden (1997) or otherwise measured by the authors.

**Table:** 

1	Basidiospores smooth	**2**
–	Basidiospores ornamented	**11**
2	Acanthophyses absent	**3**
–	Acanthophyses present	**4**
3	Basidiospores thick-walled, 23–27 × 16–21 μm; on *Rhododendron*	*** Neoaleurodiscus fujii ***
–	Basidiospores thin-walled, 18–23 × 14–19 μm; on *Quercus*	*** A. ljubarskii ***
4	Basidia with two sterigmata; basidiospores >12 µm long	*** A. canadensis ***
–	Basidia with four sterigmata; basidiospores <12 µm long	**5**
5	Generative hyphae simple-septate	**6**
–	Generative hyphae clamped	**10**
6	Acanthophyses apparently dextrinoid	*** A. dextrinoideophyses ***
–	Acanthophyses indextrinoid	**7**
7	Basidia smooth; acanthophyses yellow	**8**
–	Basidia with an acanthophysoid appendage; acanthophyses colourless	**9**
8	Gloeocystidia of two types; acanthophyses hyphoid	*** A. thailandicus ***
–	Gloeocystidia of one type; acanthophyses hyphoid, subclavate to subcylindrical	*** A. isabellinus ***
9	Texture loose; basidiospores 9–12 × 5–7.5 μm	*** A. tropicus ***
–	Texture compact; basidiospores 7–10 × 4–6 μm	*** A. bambusinus ***
10	Acanthophyses apparently dextrinoid	*** A. dextrinoideocerussatus ***
–	Acanthophyses indextrinoid	*** A. cerussatus ***
11	Acanthophyses absent	**12**
–	Acanthophyses present	**19**
12	Generative hyphae simple-septate	**13**
–	Generative hyphae clamped	**16**
13	Basidiomata discoid; basidiospores >20 µm long	*** A. amorphus ***
–	Basidiomata corticioid; basidiospores <20 µm long	**14**
14	Basidiospores <8 µm long	*** A. tenuissimus ***
–	Basidiospores >8 µm long	**15**
15	Basidiospores 12–17 × 10–15 µm; on angiosperm wood	*** A. ryvardenii ***
–	Basidiospores 8–11.5 × 6–8.5 µm; on bamboo	*** A. verrucosporus ***
16	Basidiospores >20 µm long	*** A. grantii ***
–	Basidiospores <20 µm long	***17***
17	On *Quercus*	*** Aleurocystidiellum disciforme ***
–	On gymnosperm	**18**
18	Encrusted skeletocystidia present; on *Abies*	*** Aleurocystidiellum subcruentatum ***
–	Moniliform gloeocystidia present; on *Pinus*	*** Aleurocystidiellum tsugae ***
19	Acanthophyses amyloid	*** A. botryosus ***
–	Acanthophyses non-amyloid	**20**
20	Basidiospores globose; on bamboo	*** Acanthobasidium bambusicola ***
–	Basidiospores ellipsoid; on wood	**21**
21	On gymnosperm	**22**
–	On angiosperm	**23**
22	Basidiospores 16–21 × 12–17 μm	*** A. effusus ***
–	Basidiospores 26–38 × 20–28 μm	*** A. gigasporus ***
23	Basidiomata white when fresh; acanthophyses rare	*** A. microcarpus ***
–	Basidiomata pinkish when fresh; acanthophyses abundant	**24**
24	Basidiospores 16–20 × 11–14 μm	*** A. subroseus ***
–	Basidiospores >20 µm long, >14 µm wide	**25**
25	Acanthophyses hyphoid, covered with spines at whole upper part; basidia and gloeocystidia covered with spines at basal part; basidiospores usually D-shaped	*** A. mirabilis ***
–	Acanthophyses hyphoid to clavate, covered with spines only at apex; basidia and gloeocystidia smooth; basidiospores ellipsoid	*** A. wakefieldiae ***

## Supplementary Material

XML Treatment for Aleurodiscus bambusinus

XML Treatment for Aleurodiscus isabellinus

XML Treatment for Aleurodiscus subroseus
